# CADRE: A Collaborative, Cloud-Based Solution for Big Bibliographic Data Research in Academic Libraries

**DOI:** 10.3389/fdata.2020.556282

**Published:** 2020-11-20

**Authors:** Patricia L. Mabry, Xiaoran Yan, Valentin Pentchev, Robert Van Rennes, Stephanie Hernandez McGavin, Jamie V. Wittenberg

**Affiliations:** ^1^ HealthPartners Institute, Bloomington, MN, United States; ^2^ Indiana University Network Science Institute, Indiana University Bloomington, Bloomington, IN, United States; ^3^ Big Ten Academic Alliance, Champaign, IL, United States; ^4^ Digital Strategy & Information Technology Services, University Libraries, University of Colorado Boulder, Boulder, CO, United States

**Keywords:** bibliographic big data, bibliographic research resource, libraries, open access, platform-as-a-service, reproducibility

## Abstract

Big bibliographic datasets hold promise for revolutionizing the scientific enterprise when combined with state-of-the-science computational capabilities. Yet, hosting proprietary and open big bibliographic datasets poses significant difficulties for libraries, both large and small. Libraries face significant barriers to hosting such assets, including cost and expertise, which has limited their ability to provide stewardship for big datasets, and thus has hampered researchers' access to them. What is needed is a solution to address the libraries' and researchers’ joint needs. This article outlines the theoretical framework that underpins the Collaborative Archive and Data Research Environment project. We recommend a shared cloud-based infrastructure to address this need built on five pillars: 1) **C**ommunity–a community of libraries and industry partners who support and maintain the platform and a community of researchers who use it; 2) **A**ccess–the sharing platform should be accessible and affordable to both proprietary data customers and the general public; 3) **D**ata-Centric–the platform is optimized for efficient and high-quality bibliographic data services, satisfying diverse data needs; 4) **R**eproducibility–the platform should be designed to foster and encourage reproducible research; 5) **E**mpowerment—the platform should empower researchers to perform big data analytics on the hosted datasets. In this article, we describe the many facets of the problem faced by American academic libraries and researchers wanting to work with big datasets. We propose a practical solution based on the five pillars: The Collaborative Archive and Data Research Environment. Finally, we address potential barriers to implementing this solution and strategies for overcoming them.

## Introduction: The Rise of Big Bibliographic Datasets in Research and How Libraries Struggle to Meet Demands

Big bibliographic datasets hold promise for revolutionizing the scientific enterprise when combined with state-of-the-science computational capabilities (Fortunato et al., 2018). Yet, hosting proprietary and open big datasets poses significant difficulties for libraries, both large and small. Libraries in the United States are central and necessary institutions in the acquisition, preservation, and dissemination of big bibliographic datasets for several reasons. Acquiring data for continued research use depends on a technical and legal framework that has been long-established in research libraries (Li et al., 2019). Furthermore, U.S. libraries are uniquely positioned to facilitate reproducible research due to their services and infrastructure supporting assessment, preservation, and provenance (Tripathi et al., 2017). Barriers to hosting include cost and expertise in infrastructure hosting (especially for cloud hosting), proprietary access restrictions (i.e., data security), data cleaning, and other data custodial tasks, data updates, and maintenance, and enabling big data analytics (with appropriate security and sharing), including enabling analytics for patrons without advanced programming ability. As a result, the user base for these datasets is limited to individual researchers or large and well-funded academic libraries with the resources and technical expertise to host big data. This is true for both proprietary and open bibliographic data. This problem is pervasive; each individual academic library in the U.S. faces the same conundrum. Libraries are increasingly acquiring big bibliographic datasets in recognition of their research patrons’ needs without a robust hosting solution. This means that the datasets cannot be used to their full research potential. Internationally, some countries with more centralized higher education infrastructure have made advances in developing shared resources that begin to address these problems.^b^ ^,^ ^c^ In the United States, however, many libraries choose not to invest in acquiring big datasets, even open ones, due to the lack of resources available to enable access in their campus computing environment.

## Five Pillars for a Big Bibliographic Data Hosting Solution: Community, Access, Data-Centric, Reproducibility, and Empowerment

We suggest that the answer may lie in CADRE^a^ a cloud-based platform for text and data mining, which could provide sustainable, scalable, and standardized data and analytic services for open and proprietary big bibliographic datasets.

**FIGURE 1:**
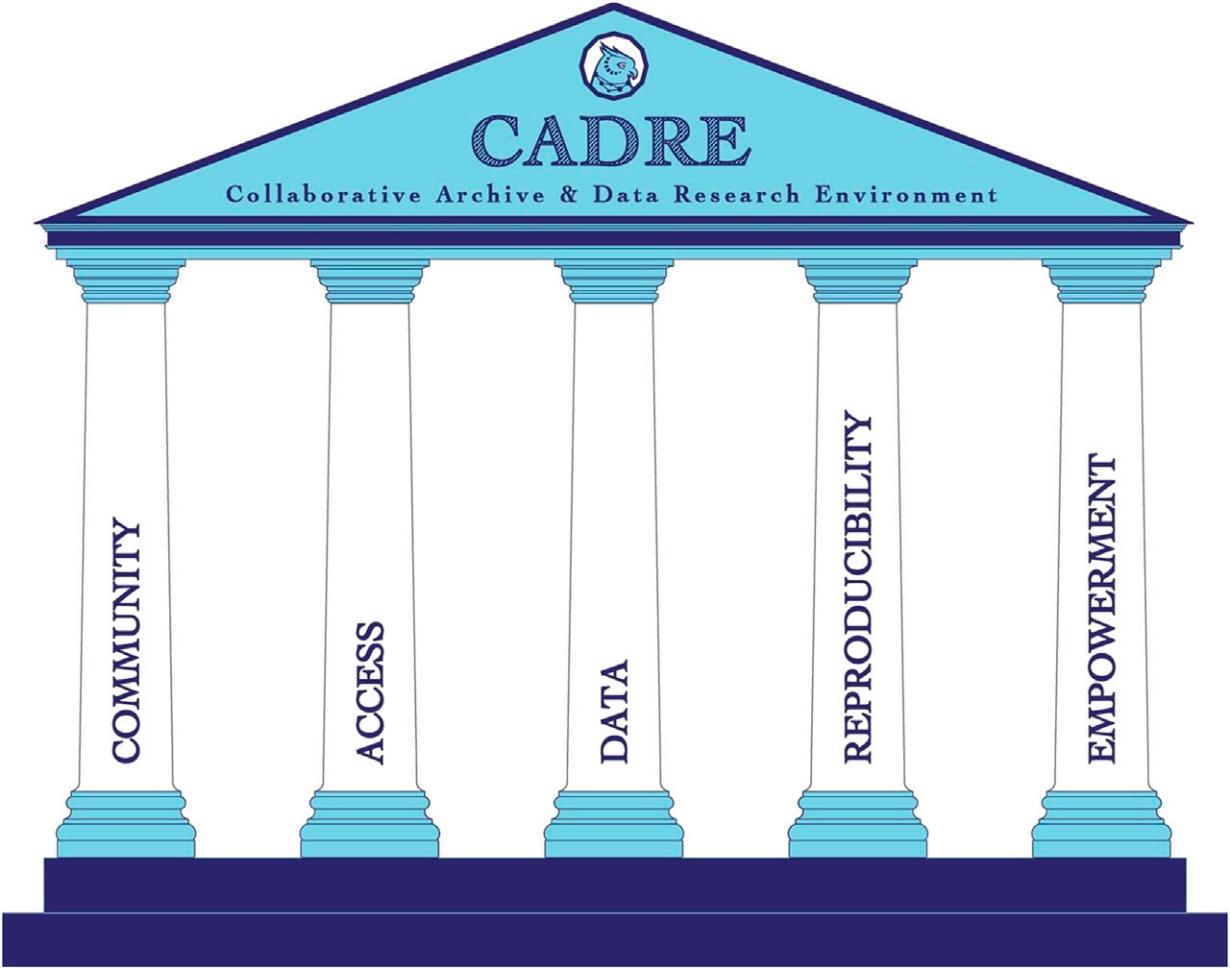
The five pillars of CADRE.

**FIGURE 2:**
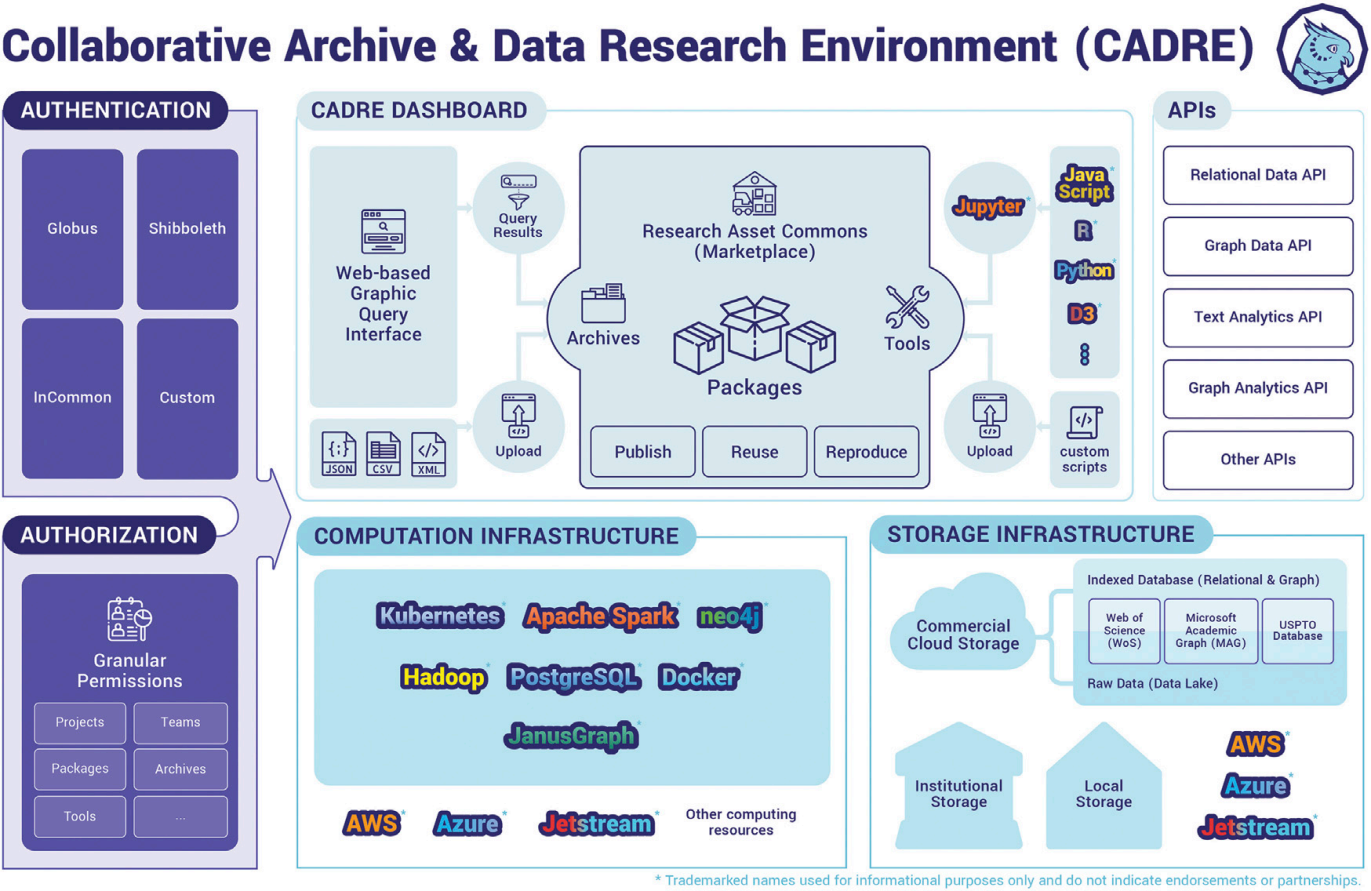
Envisioned blueprint of the CARE platform.

The proposed solution rests on five pillars, **C**ommunity, **A**ccess, **D**ata-Centric, **R**eproducibility, and **E**mpowerment (as seen in Figure 1), and is informed by previous work including the National Opinion Research Center (NORC)^d^ (Birkle et al., 2020) and National Institute of Standards and Technology’s (NIST)^e^ (Christenson, 2011), NORC Data Enclave^f^ (Edwards, 1999) and the HathiTrust Digital Library (Lane and Shipp, 2008; Christenson, 2011). A schematic of the proposed solution is shown in Figure 2.

### Community

Two distinct communities are essential to the success of a cloud-based platform solution: 1) A community of libraries, researchers, educational institutions, industry partners, and a technical team with relevant expertize should be formed to jointly take on the development, governance, and sustainability of the platform. 2) A community of platform users (researchers, librarians, administrators) is needed to test out the platform and give input on the features and functionality, data-hosting choices, and to add value through sharing of their own platform-derived research assets.

#### Library-Industry-Technical Team Collaboration

There is a strong theoretical basis underpinning the role of academic libraries supporting data and text mining, including their roles as data quality hubs on campus (Giarlo, 2013). Provisioning large datasets is a modern incarnation of the collection building and stewardship with which libraries have always been charged (Friedlander and Adler, 2006). Libraries have long recognized that collaborative approaches to acquisition, technical services, and research infrastructure exponentially increase their buying power. For example, the Big Ten Academic Alliance’s (BTAA) library objective is “*optimizing student and faculty access to the combined resources of our libraries; maximizing cost, time, and space savings; and supporting a collaborative environment where library staff can work together to solve their mutual problems*.”^g^ The BTAA consortium consists of 14 academic libraries keenly affected by the big dataset hosting problem, and the BTAA is well-positioned to play a strong role in shaping the cloud-based platform solution we describe here. A technical team with experience in hosting, cleaning, updating, securing, and otherwise setting up and running big data hosting and computing environments is also needed. Ideally, the technical team would have experience in hosting proprietary big bibliographic datasets and have an existing relationship with the vendors.

Libraries and industry should come together to share both the financial and decision-making burdens that come with hosting the platform, as well as the burden of staffing and overseeing the person power to build, implement, and maintain the platform. Many benefits would convey to all parties concerned. For example, by uniting in a consortium, academic libraries large and small can gain bargaining power when purchasing from proprietary vendors (e.g., Web of Science) or cloud-computing and storage-service providers (e.g., Microsoft Azure, Amazon Web Services). The libraries would see substantial cost savings through a single, jointly-financed technical team vs. duplicating teams (and costs) at each institution. Another benefit would be the time saved by not having to oversee and manage so many teams. Existing local and national efforts to build infrastructure in support of data-intensive research, like XSEDE,^h^ often do not offer services or architecture that accounts for the nuanced, disparate licensing requirements of many large, proprietary datasets. This key feature of the CADRE solution differentiates it from similar platforms.

#### Community of Platform Users

There are many constituencies to take into account when providing access to big data and related services for research: researchers who rely on bibliographic datasets for their research careers (e.g., researchers in informetrics, scientometrics, library science, digital humanities, and related fields of study); librarians who use the big bibliographic datasets to understand their patrons’ needs and inform their decisions about managing their collections and services; and others, including the general public.

A shared hosting solution would not only benefit these users by enhancing their individual research projects, but would greatly enhance the productivity of the field by helping these constituencies find one another and enhance the comparability of their research through the use of shared standards (which they would be called on to create), shared data (including ability to use the same versions), and shared data custodial tasks and costs. Users would also be able to communicate directly with one another through a Research Asset Commons section of the platform where research assets, such as results, code, algorithms, workflows, visualizations, data, and analytics (including appropriate software versions and specific computing environments), could be shared to ensure reproducibility.

For research libraries, there is potential benefit in using the hosted bibliographic datasets for service enhancements. For example, citation analysis can be an important tool in evaluating journal relevance (Gureev and Mazov, 2015) and in collection development (Edwards, 1999; Belter and Kaske, 2016). Access to bibliographic and other text-based datasets would enable academic libraries with appropriate subscriptions to provide a high level of analysis service to data-intensive researchers without the requirements of local expertize or infrastructure. Library-licensed data with nuanced and stringent licensing policies for acceptable use could be made available on the platform. Having a technical team with experience and expertise in working with bibliographic data vendors and establishing infrastructure and business practices that ensure compliance with data agreements would be essential.

### Access

Research libraries almost universally struggle to grant access to a corpus of large datasets to their patrons at scale. Often, libraries are forced to decide which dataset to choose from among the many in demand by researchers. Even for those libraries that can afford large proprietary datasets (i.e., Web of Science, Birkle et al., 2020), needs such as data security requirements and assurance that users abide by the terms of use of the purchase agreement can present a significant barrier. With the advent of the open data movement, many large datasets are now available—Microsoft Research’s Microsoft Academic Graph (MAG, Wang et al., 2019) and U.S. Patent and Trademark Office data^i^ are two popular bibliographic datasets that are free of charge. However, MAG is only available in raw text, delivered to the vendor’s own cloud storage. There are significant infrastructure and data custodial tasks required to make the data usable. Many users who do have access to large datasets clean and prepare only a portion of the data—the small part they need for their own work. Moreover, each user may clean a different version of the data or may do so using different algorithms. The result is a patchwork of different versions of the data, even in cases where researchers started with the same raw data. This makes comparison across research studies difficult and hinders reproducibility. By developing a single platform for sharing data, not only are the data custodial tasks handled only once, more importantly, they are standardized, resulting in all users working with the same version of the cleaned data.

We advocate for a free tier of access to the open datasets hosted on the platform for public use. With regard to access to commercially licensed datasets, a platform would open up access to libraries (and their patrons) that already purchased or want to host one or more large proprietary dataset but do not have expertise or infrastructure to support or maintain the data. A key aspect of accessibility is affordability. By sharing costs across the library and industry partners, access to big datasets will become cheaper for partner libraries that are leveraging their collective bargaining power. This strategy will also make big bibliographic datasets more affordable for small libraries that would not otherwise be able to afford them (i.e., their purchase and/or their support and maintenance). Moreover, a cloud-based computing solution would allow users to consume computing resources on a pay-as-you-go basis, which would be especially valuable to users who are affiliated with smaller institutions and who lack the budget for large computing resources.

Data security is central to any licensed big dataset. In order to grant access appropriately while maintaining stringent controls that protect data security, we propose a federated login system. Leveraging universities' existing single-sign-on systems, access to the licensed big datasets can be federated, granting access based on university credentials and mapping them to the university’s permissions.

### Data-Centric

For the long-term sustainability of the platform, the relevant parties would need to identify and prioritize the datasets that are in-demand and serve the interests of the libraries’ patrons and are also appropriate for cloud-based big data solutions. We believe a mutually beneficial agreement between libraries, users, and the industry (e.g., vendors of proprietary datasets) can be negotiated for the betterment of all parties.

Hosting a small number of centralized datasets allows for the data infrastructure to be optimized to meet technological challenges which individual institutions are ill-equipped to handle on their own due to lack of financial resources and expertise. Big data is generally defined in four dimensions (Yin and Kaynak, 2015): *volume* (size of the data), *velocity* (continuous flow of incoming and/or updated data), *variety* (diversity in data formats and usage), and *veracity* (data quality). Bibliographic data sets present unique challenges in all dimensions and require special technical solutions.

A centralized data model is crucial for solving *volume* challenges (Foster et al., 2008). Shared cloud storage avoids duplicate data storage and multiple versions of the same data. Combined with a fully featured data analytical environment, it adheres to a more efficient model of “Moving Computation to Data” (Zaharia et al., 2010), minimizing large volume data movement. The centralized volume efficiency also allows specialized database designs. For example, higher order citation analyses are frequently needed in bibliographic research, but they present a major computational challenge for traditional solutions such as relational databases. For this type of problem, graph databases provide an efficient alternative (Angles, 2012). In general, a systematic solution should offer the data in different formats and database designs, each optimized for specific types of analysis.

In terms of *velocity*, many large bibliographic datasets are prone to change over time as a result of correcting the dataset, augmenting the dataset with newly acquired data, and even adding new fields as the dataset evolves. These modifications, while intended to be beneficial, result in versioning problems where different labs end up using different versions of the data from one another or within a single work group over time. This can hinder interpretation of results across studies and complicate reproducibility efforts. A shared data hosting platform can mitigate such problems by ensuring that all members access the same version of the data with the same schedule of updates.

With respect to data *variety*, consumers may differ in their technical capabilities and desired research outputs and a solution should be able to adapt accordingly. A platform capable of hosting multiple datasets under common standards makes comparative and integrative studies possible. Additional local computational infrastructure can also complement the centralized cloud environment (Goyal, 2014). Flexible and efficient computational resource allocation is essential for ensuring that users across a wide spectrum of needs and preferences have a satisfactory experience (i.e., it should be easy to use and satisfy their goals) while minimizing computational cost overall. To complement the centralized databases, user generated data should be an integral part of the ecosystem, allowing flexible incorporation of different data sources.

Data *veracity* is the degree to which data is accurate, precise, and trusted. Sharing the cost will permit high-quality data parsing, cleaning, and enrichment at very little cost while greatly increasing the value and acceptance of the data. One example is author disambiguation, which important bibliometric research depends upon. However, no widely agreed standard exists for determining if two authors with the same or similar name in different datasets are in fact the same author or two different authors. A platform with active user feedback and a dedicated technical team can catalyze the formation of such data standards, further enhancing the platform’s value.

### Reproducibility

A big advantage of a single, shared data hosting platform would be data version control across library access points, making them much more comparable across studies and reducing barriers to reproducibility and replicability. Moreover, a platform would add great value if it could also be designed to encourage the community to set standards for Research Assets (defined above). The use of globally unique Digital Object Identifiers (DOIs) would ensure reproducibility and replicability of results, as well as data provenance for every stage of their transformation. The key to making research reproducible is documenting and archiving relevant data, analyses, software, and computing environments. Use of virtual machines and container technologies could ensure that data and software version control are documented automatically. Each user should be empowered to choose and modify a default level of sharing as well as tailored sharing for any research asset they desire. Levels of sharing should be: no sharing (research asset is private), sharing with specific named individuals or groups (user grants sharing permission on a per research asset and/or per person basis), public sharing (selected research assets or all assets are shared publicly). A platform using virtual machines (VM) and container technologies could be set up to ensure full reproducibility by capturing the exact versions of data, software, libraries, and tools used for each analysis and would also enable reuse of these resources with or without modification.

Computational reproducibility sets the foundation for higher level of scientific rigor, such as proper treatment of misinformation and biases in model and data, or statistical reproducibility in general (Stodden, 2013). A platform with multiple datasets and a wide researcher user base can help to promote reproducibility and comparison studies, best practices in data driven research, and facilitate the formation of a more open and rigorous research community.

### Empowerment

The aim of any data sharing solution should be to allow researchers to access big data in the form most appropriate to the user. Therefore, a platform with the capability to serve the user in graph-based, relational, flat tables, and native formats, which can be automatically parsed according to computational needs or manually selected by the user, would be ideal. Moreover, the solution should provide access to supercomputing resources at users’ own research institution (i.e., through single sign on or other appropriate identity verification). It is important to offer users multiple tiers and the option to bring their own compute resources. This flexibility would create opportunities for users who otherwise would not have big data analytical capabilities, while lowering costs for those who already possess computational resources. All platform functions should access data and tools via Application Programming Interfaces (APIs). The platform should host analytic tools and use appropriate data processing/querying software and be designed to permit access to private cloud and local compute resources. Providing a coding environment which is directly accessible through the web interface, where researchers well-versed in scripting languages would be able to access resources directly or through an API to engage big data query and analytics services,^j^ would be ideal. Utilizing native cloud visualization systems could ensure users are given an easy and integrated way to visualize results.^k^

## Discussion

In this article we have described the ubiquitous problem that U.S. researchers and the academic libraries they patronize face in the era of big datasets: individual libraries lack the human and financial resources to host large open and commercial bibliographic datasets. We have presented a detailed potential solution to the problem resting on five pillars: *C*ommunity, *A*ccess, *D*ata-Centric, *R*eproducibility, and *E*mpowerment. Here, we propose a path forward to realizing such a solution and identify possible barriers.

One significant barrier to the proposed solution is the financing of the platform. The suggested solution attempts to bring together a broad group of partners including federal funding agencies, such as the Institute for Museum and Library Services (IMLS), which has awarded a grant to the project. Other partners include academic libraries, especially those in consortia like the BTAA, who recognize they have much to gain by leveraging their shared interests, finances, and bargaining power. Finally, data vendors such as Web of Science Group and Microsoft Research, have a vested interest in the development of such a platform as a way to attract new users from institutions that cannot otherwise afford to invest in data without a hosting site and infrastructure. Ultimately, a platform based on the proposed pillars will greatly facilitate the use of large datasets while reducing costs to all.

This is because the proposed platform, though more expensive than what any individual library would likely develop as a single-institution solution, becomes far less expensive when the cost is divided by the number of participating members. Moreover, the cost decreases and the benefits remain the same or increase as the number of members increases. So, while obtaining financial support is always a delicate and often difficult process, we see a bright future given the potential benefits to all parties.

A second barrier is the possibility that such a platform would not be adopted by researchers and librarians. We have talked with many people in both groups and have keenly felt the need for such a platform. We propose many enticements for these groups starting with access to the large datasets with a variety of computational options, including cloud or local computing resources, through web programming environments such as Jupyter notebooks and RStudio. The flexible choice of data formats and database designs, would enable, for example, multi-hop graph analytics through a state-of-art graph database backend. Other features noted above include a reproducible workspace (virtual machine) for each researcher and a shared workspace for sharing research assets with others (i.e., advertising one’s own work), as well as for reusing others' research assets, such as data, visualizations, code, etc.—with appropriate permission and attribution. We have also suggested a Graphical User Interface (GUI) to facilitate query building for those without programming skills. Such a GUI can enable easy extraction of bibliographic data for administrative purposes such as journal cost analysis of faculty publications by institutions. Enticements are probably insufficient without intentional promotion of the platform, such as at conferences and meetings of researchers and librarians. We think that offering fellowships (early access to data and cloud computation as well as technical support) to entice early adopters would be key. These users would be ideal for offering suggestions on the features and datasets to include as well as for identifying problems and bugs. Offering workshops and webinars to help the targeted communities become aware of the tool and learn how to use it is another strategy to help ensure a sufficient user base. Lastly, reducing access barriers through a free public access tier would make sure no one is left out.

A third threat to the proposed platform is sustainability. Obtaining one-time resources to build a platform is one thing. Finding ongoing support for platform maintenance, data updates, ongoing security, technical support, and addition of new datasets, requires a sustainability plan. We believe that the benefits are so great that individual libraries would pledge annual contributions that collectively would be adequate to maintain and even enhance the capabilities of the platform over time. This membership model has proven successful for other library initiatives (e.g., Christenson, 2011).^l^ The Data Curation Network,^m^ for example, has effectively established a network of expertise across research libraries that enables individual libraries to focus their investment on an area of pressing need for their community, but reap the benefits of curatorial expertize for other data types (Johnston et al., 2018). The Lyrasis sustainability analysis for this project (Arp et al., 2020), based on their “*It Takes a Village: Open Source Software Models of Collaboration and Sustainability*”^n^ project, has significant implications for the sustainability of library-based collaborations such as the one we propose. The research library community in the United States has also demonstrated willingness to contribute to shared efforts that address a common need, such as adding value to library collections. The Collections as Data effort (Padilla et al., 2019), a framework for enabling computational access to library digital collections, is one such example.

The barriers to implementing a cloud-based, data-sharing platform are significant, but the merits of one are such that the concerned parties will come together to create an operational platform that will offer researchers a superior solution to big bibliographic data access at a lower cost. Developing a platform based on the CADRE pillars has the potential to simultaneously solve a significant problem for academic libraries and researchers, drastically reduce costs and effort, increase efficiency, improve quality, and enhance reproducibility. With the proper investment and a collaborative effort, the concept for the platform described above will be the solution that researchers and libraries need.

## Data Availability Statement

The original contributions presented in this article are derived from our experience with the CADRE project. Additional information about the project is located at: http://doi.org/10.26313/rdy8-4w58. Inquiries can be directed to the corresponding author.

## Author Contributions

JW, PM, VP, XY, and RR contributed to the conception and design of the CADRE Project. PM led the drafting of the manuscript. SM created the pillars concept. All authors contributed to the conception and development of the manuscript and the pillars, wrote or developed sections of the manuscript, contributed to manuscript revision, read, and approved the original and revised versions.

## Funding

The authors are supported to work on a project to develop the Collaborative Archive & Data Research Environment (CADRE) as well as disseminate information about the project, including the writing of this manuscript. The support comes from a grant from the National Leadership Program at the U.S. Institute of Museum and Library Services (IMLS; *Shared BigData Gateway for Research Libraries,* award number LG-70-18-0202-18). In addition, cost share for the project is contributed by: Microsoft Research, Web of Science Group, The Big Ten Academic Alliance Library Initiatives, the Pervasive Technology Institute at Indiana University, Indiana University Network Science Institute, Indiana University Libraries, Michigan State University Libraries, Ohio State University Libraries, Penn State University Libraries, Purdue University Libraries, Rutgers University Libraries, University of Iowa Libraries, University of Michigan Libraries, and University of Minnesota Libraries. Support for CADRE also comes from membership fees from our members: all of the above named libraries plus the University of Maryland Libraries.

## Conflict of Interest

The authors wish to declare a potential conflict of interest with one of the journal Associate Editors, Kuansan Wang. Kuansan helped secure funding from his employer, Microsoft Research. The funds were used to provide travel scholarships to CADRE Fellows to attend and present on their CADRErelated work at scientific meetings.

## Footnotes

b One example is the Competence Center for Bibliometrics: http://www.forschungsinfo.de/Bibliometrie/en/index.php?id=impressum.

c See also, EOSC Declaration: https://ec.europa.eu/research/openscience/pdf/eosc_declaration.pdf.

d National Opinion Research Center (NORC): https://www.norc.org/About/Pages/default.aspx.

e National Institute of Standards and Technology (NIST): https://www.nist.gov/about-nist.

f NORC Data Enclave: https://www.norc.org/Research/Capabilities/Pages/data-enclave.aspx.

g Big Ten Academic Alliance Libraries: https://www.btaa.org/library/libraries

h XSEDE: https://www.xsede.org/.

i United States Patent and Trademark Office Bulk Data Products: https://www.uspto.gov/learning-and-resources/bulk-data-products.

j Integrated Jupyter and Databricks notebooks could provide access to resources like Spark, R, Python, Scala, or SQL, SPARQL, Cypher and Gremlin.

k QuickSight and Power BI are two cloud-based visualization systems that could be used.

l See also, OCLC: A Nonprofit, Membership Cooperative: https://www.oclc.org/en/about/finance.html.

m The Data Curation Network website: https://datacurationnetwork.org/.

n “It Takes a Village” website: https://www.lyrasis.org/programs/Pages/IMLS-OSS.aspx.
